# Novel Insights into the Influence of Soil Microstructure Characteristics on the Migration and Residue of Light Non-Aqueous Phase Liquid

**DOI:** 10.3390/toxics11010016

**Published:** 2022-12-24

**Authors:** Xiaodong Li, Qian Zhang, Xueli Zhang, Jialun Shen, Zongquan Sun, Fujun Ma, Bin Wu, Qingbao Gu

**Affiliations:** 1 Technical Centre for Soil, Agriculture and Rural Ecology and Environment, Ministry of Ecology and Environment, Beijing 100012, China; 2 State Key Laboratory of Environmental Criteria and Risk Assessment, Chinese Research Academy of Environmental Sciences, Beijing 100012, China

**Keywords:** diesel, soil particle and pore, statistical analysis, key factor

## Abstract

Understanding the influence of soil microstructure on light non-aqueous phase liquids (LNAPLs) behavior is critical for predicting the formation of residual LNAPLs under spill condition. However, the roles of soil particle and pore on LNAPLs migration and residue remains unclear. Here, the experiment simulated an LNAPLs (diesel) spill that was performed in fourteen types of soils, and the key factors affecting diesel behavior are revealed. There were significant differences between fourteen types of soils, with regard to the soil particle, soil pore, and diesel migration and residue. After 72 h of leakage, the migration distance of diesel ranged from 3.42 cm to 8.82 cm in the soils. Except for sandy soil, diesel was mainly distributed in the 0–3 cm soil layer, and the residual amounts were 7.85–26.66 g/kg. It was further confirmed from microstructure that the consistency of soil particle and volume of soil macropores (0.05–7.5 μm) are important for diesel residue in the 0–1 cm soil layer and migration distance. The large soil particles corresponding to 90% of volume fraction and volume of soil mesopores (<0.05 μm) are key factors affecting diesel residue in the 1–3 cm soil layer. The result helps to further comprehend the formation mechanism of residual LNAPLs in the soil.

## 1. Introduction

Chemical spill caused by tank bottom corrosion, pipeline damage, or facility explosion has increased the frequency and harm degree of soil pollution incidents, with the spill of petroleum products especially being the most frequent [1,2,3]. Petroleum products (e.g., diesel) are water insoluble or sparingly water soluble, which belongs to light non-aqueous phase liquids (LNAPLs) [4]. After the spill of petroleum products, it will migrate downward at the soil surface under the action of gravity. During the migration process, petroleum products could accumulate at different locations underground, and form residual states such as accumulation areas or lenses of NAPLs, resulting in the long-term pollution of soil and groundwater environment [5,6]. 

The formation process of residual LNAPLs is affected by the characteristics of LNAPLs, environmental conditions, and soil structure (e.g., soil particle size, soil pore size, and spatial heterogeneity) [7,8,9,10]. Under spill conditions of specific LNAPLs, soil structure is the main factor to control the formation of residual LNAPLs [7,11]. Therefore, the quantitative characterization of soil structure is conducive to understanding the influence degree and mechanism of different types of soils on the formation of residual LNAPLs. Due to different environmental factors, soil properties vary greatly in different regions, which resulted in the disparity of soil structure characteristics in China [12,13,14]. However, to the best of our knowledge, there are no relevant studies on the systematic quantitative analysis of soil structure characteristics in the large-scale region of China. 

In recent years, a few studies have explored the impacts of soil structure characteristics on the formation process of residual LNAPLs under spill condition [15,16,17,18]. Saripalli et al. characterized the relationship between the soil pore size and spatial distribution of residual LNAPLs by the interfacial tracers technique and morphological method [16]. Soil macropores and mesopores were the key factors affecting the migration rate of fluid, and the number of soil micropores controlled the residual amount of fluid in the soil [15]. With the increase in specific pore surface area, the migration rate of LNAPLs in porous medium tended to decrease [10,19]. It has been reported that although two types of soils had the same soil porosity and organic matter content (OM), soil with high clay content possessed more micropores, resulting in a higher residual amount of LNAPLs in the soil [20]. Moreover, soil properties are largely determined by soil weathering, which thus affected soil classification order [21]. For example, the Vertisols are highly fertile due to their high clay content, but most of the water content is inaccessible to plants. Entisols are the most recently developed soils abounding illites and kaolinitic minerals or well-crystalline oxides. Alfisols have a high content of Al and Fe oxides, good structure, and typical slightly acidic activity [22]. Golia et al. [23] reported that high regression coefficients were observed between soil pH and potentially toxic elements concentrations in Alfisols, reflecting that soil pH is the dominant characteristic influencing potentially toxic elements. In Vertisols, clay content proved to be the most important parameter influencing potentially toxic elements concentrations levels. Therefore, the soil classification order-dependent diversity can be assumed to lead to the different formation processes of LNAPLs in soils. However, most previous studies have been restricted to the impact of a single soil structure characteristic on the formation process (migration and residue) of residual LNAPLs in the soil [24]. Due to the lack of statistical quantitative analysis, the key factors of soil structure affecting migration and residue of LNAPLs under spill condition remained unclear. 

In this study, fourteen types of soils in China were selected from surface soils of different geographic regions, and further identified the distribution characteristics of soil particles and pore size using several characterization methods. Diesel is chosen as the typical petroleum pollutant due to being widely used in society. Then, the simulated accidental spill was performed in fourteen types of soils to investigate the migration distance and vertical distribution of diesel. Based on soil structure characteristics in the large-scale region of China, the multivariate linear regression of the migration distance and residual amount of diesel with soil structure characteristics are established through the statistical product service solutions (SPSS) statistical analysis, and revealed the sensitive factors which affect the migration distance and residual amount of diesel. The result will be a benefit to further comprehending the formation process and mechanism of residual LNAPLs in the soil.

## 2. Materials and Methods

### 2.1. Soil Samples

Fourteen types of soils were collected from surface soils (0–20 cm) distributed in different geographic reigns across north to south China: Heilongjiang and Jilin in northeast China; Hebei in North China; Henan and Hunan in central China; Zhejiang, Jiangsu, Jiangxi, and Shandong in east China; Shaanxi and Qinghai in northwest China; Hainan in south China; Guizhou and Chongqing in southwest China. All soil samples were naturally air-dried indoors, passed through a 2 mm sieve, and stored in plastic containers at room temperature. Basic physiochemical properties including pH, OM, and cation exchange capacity (CEC) of soil samples were analyzed by routine methods [25,26]. The soil pH was measured in a 1:2.5 (*w*/*v*) soil/water suspension after 1 h equilibration using a SevenCompact™ S210 pH Meter (Mettler-Toledo). The soil OM was measured by the potassium dichromate volumetric method. The ammonium acetate method was used to measure the CEC. Soil particle size distribution was determined by a laser particle size analyzer. Soil pore size distribution was characterized by a mercury piezometric method. 

The Characteristic parameters included Dv10, Dv30, Dv50, Dv60, Dv90, Cu, and Cc [27,28,29]. Dv10, Dv30, Dv50, Dv60, and Dv90 represent the soil particles corresponding to 10%, 30%, 50%, 60%, and 90% of volume fraction, respectively. The nonuniform coefficient (Cu = Dv60/Dv10) is defined as the uniformity of soil particle size distribution. The curvature coefficient (Cc = (Dv30)^2^/Dv10∙Dv60) reflects the overall shape of soil particle size distribution curve. The consistency and diameter distance are the characteristic parameters used to describe the deviation and width of soil particle size distribution curve, respectively. 

### 2.2. Experimental Device

A one-dimensional soil column was used to simulate the migration of diesel in the soil under spill condition. The soil column (238.5 cm^3^) was obtained by filling a cylindrical glass tube with a length of 25.0 cm and a 4.5 cm inner diameter, which was lined with quartz flakes in the lower part and connected to the atmosphere in the upper part. Specifically, 1 cm-thick soil was weighed in batches according to actual soil bulk density (a total of 15 cm soil column), then slowly poured and compacted into a cylindrical glass tube. The soil column is composed of 2 cm coarse quartz sand (20–40 mesh), 15 cm test soil, and 1 cm fine quartz sand (40–70 mesh) from top to bottom. Coarse quartz sand could prevent the loss of diesel due to splashing from the spill process, and also make diesel evenly distributed on the soil surface.

### 2.3. Experimental Procedures

To simulate the instantaneous diesel spill under no rainfall condition, 10 mL of diesel was dumped above the soil column at one time. Then, the migration distance (S) of diesel front was observed and recorded at regular intervals to analyze the dynamic process of diesel migration. The 5.0 g soil sample was withdrawn using a five-point sampling method in each soil layer and mixed to guarantee the homogeneity of the samples. The mixed soil samples were collected and determined the residual amount of diesel (*Y*). In the pre-experiment, three types of soil-filled columns (Jiangxi red earth, Heilongjiang black soil, and Shaanxi loessal soil) were selected, and the samples were taken at 1 h, 5 h, 12 h, 1 d, 2 d, 3 d, and 5 d after the spill occurred, respectively. By analyzing the variation of diesel residue in the soil, the steady state of residual diesel was achieved in 3 days. The migration experiment was conducted at a constant temperature of (20 ± 2) °C and in triplicate.

### 2.4. Analytical Methods

Take 2.00 ± 0.01 g of contaminated soil and put it into a polytetrafluoroethylene centrifuge tube. Add 5 mL of dichloromethane for ultrasonic extraction for 30 min, and then centrifuge at 3000 rpm for 15 min. The method for determining the concentration of diesel refers to the previous studies [26]. A DB-5 capillary column was used, and the carrier gas was helium (purity, 99.999%). The flow rate was 1.5 mL/min. The oven temperature of GC was programmed from 50 °C (2 min) to 250 °C at 8 °C/min (3 min). The temperatures of the injector, ion source, and transfer line were set to 250 °C, 230 °C, and 280 °C, respectively.

## 3. Results and Discussion

### 3.1. Basic Physiochemical Properties and Structure Characteristics of Soils

#### 3.1.1. Basic Physiochemical Properties of Soils

Fourteen types of soils were collected from different geographic regions in China with a wide distribution range. Soil classification of the studied pedons according to the U.S. Soil Taxonomy is conducted and soils were classified into 4 orders, including Mollisols (Heilongjiang and Jilin black soil), Alfisols (Shaanxi loessal soil, Hebei and Henan fluvo-aquic soil, and Hunan red earth), Entisols (Shandong fluvo-aquic soil, Qinghai gray desert soil, and Chongqing purple soil), and Ultisols [30]. The basic physiochemical properties were quite different in various regions (Table 1). Guizhou yellow soil is a strongly acidic soil with the lowest pH value (3.96), Shaanxi loessal soil has the highest pH value of 8.76, and most of the soils are weakly alkaline. The organic matter content of Jilin black soil was the highest at 47.90 g/kg, and the cation exchange capacity of Hainan sandy soil was the lowest (1.92 cmol/kg). The soil bulk density in fourteen geographic regions was between 1.295 g/cm^3^ (Jilin black soil) and 1.873 g/cm^3^ (Hainan sandy soil). The soil clay content in Heilongjiang was the highest at 43.3%. This great variation in the basic physiochemical properties of soils was attributed to the corresponding differences in terrain, climate, vegetation, and soil age [31]. According to soil particle size distribution [32], fourteen types of soils were divided into sandy soil, sandy loam soil, sandy clay loam soil, clay loamy soil, and sandy clay soil. 

#### 3.1.2. Characteristic Parameters of Soil Particle Size Distribution

Soil particle size distribution is one of the factors affecting the migration distance and residual amount of pollutants. As shown in Table 2, the characteristic parameters of soil particle size distribution varied greatly. The average particle size (D[3,2] and D[4,3]) of Hainan sandy soil was the largest (15.53 μm and 303.27 μm), while it was lower in Guizhou yellow soil and Hunan red earth. In all, the average particle size of fourteen types of soils followed by sandy soil > sandy clay loam soil > clay loamy soil. The diameter distance and consistency of Zhejiang paddy soil were 15.38 and 3.97, respectively, indicating that the soil particle size of paddy soil in China showed great heterogeneity [33]. Notably, there was an autocorrelation between characteristic parameters of soil particle size distribution (Table S1), so it is necessary to conduct a multicollinearity diagnosis of characteristic parameters of soil particle size distribution (Table S2). The SPSS software was used for principal component analysis on characteristic parameters to obtain the main components containing all information on soil particle size distribution (Table S3). The two principal component factors were obtained (*F*_1_ and *F*_2_), and their characteristic roots were determined to be 8.987 and 2.213, respectively. The variance contribution rates to each variable are 74.893% and 18.445%, respectively, which can explain 93.337% of soil particle size distribution.

*F*_1_ = 0.1395*Z*_1_ + 0.1264*Z*_2_ + 0.1111*Z*_3_ + 0.1069*Z*_4_ + 0.1107*Z*_5_ + 0.1115*Z*_6_ + 0.1004*Z*_7_ + 0.1023*Z*_8_ + 0.1017*Z*_9_ − 0.0662*Z*_10_ + 0.0939*Z*_11_ + 0.0777*Z*_12_ (1)

*F*_2_ = 0.1416*Z*_1_ + 0.0786*Z*_2_ + 0.0129*Z*_3_ − 0.007*Z*_4_ + 0.0121*Z*_5_ + 0.0173*Z*_6_ − 0.0305*Z*_7_ − 0.0133*Z*_8_ − 0.0129*Z*_9_ + 0.0612*Z*_10_ + 0.5167*Z*_11_ + 0.4945*Z*_12_ (2)

#### 3.1.3. Characteristic Parameters of Soil Pore Size Distribution

**Figure 1:**
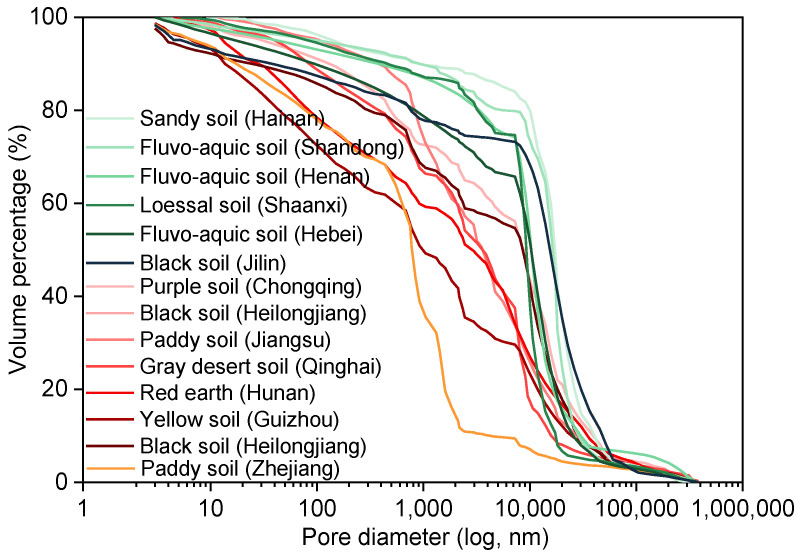
Soil pore size distribution curves of fourteen types of soils.

The curves of soil pore size distribution were obtained according to the measurement results of mercury piezometric method [34,35]. As shown in Figure 1, the pore volume percentages of fourteen types of soils decreased gradually with the increase in pore size. When the pore size ranged from 0.5 μm to 30.0 μm, there was an obvious decreasing trend in the curves of soil pore size distribution, which indicated that the soil pore size was mainly concentrated at 0.5–30.0 μm (Table S4). According to the curves of soil pore size distribution, the pore volume of different soil pore sizes (>75.0, 30.0–75.0, 7.5–30.0, 0.5–7.5, 0.05–0.5, and <0.05 μm) were calculated (Table S5). The pore volumes of soil pore size (7.5–30.0 μm) of Henan fluvo-aquic soil, Hainan sandy soil, Shaanxi loessal soil, Chongqing purple soil, Shandong fluvo-aquic soil, Heilongjiang black soil, Hebei fluvo-aquic soil, and Jilin black soil were mainly distributed from 0.09 cm^3^/g to 0.17 cm^3^/g. The pore volumes of Qinghai brown soil and Zhejiang paddy soil in the range of 0.5–7.5 μm were 0.0965 cm^3^/g and 0.0956 cm^3^/g, respectively. Most of the soil pore size of Jiangsu paddy soil, Jiangxi red earth, Hunan red earth, and Guizhou yellow soil was lower than 7.5 μm, and the pore volumes were 0.0243, 0.052, 0.0462, and 0.0317 cm^3^/g, respectively. This phenomenon was due to the fine-grained soils with high content that could easily enter the interior of the mesopores, which reduced the connectivity of soil pores and formed fine pores [36].

### 3.2. Migration Distance of Diesel in Fourteen Types of Soils

**Figure 2:**
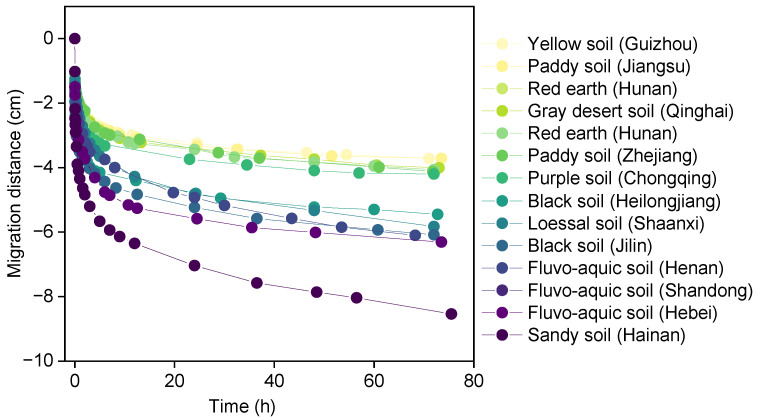
Migration kinetics of diesel in fourteen types of soils.

With the leakage of 10 mL diesel on fourteen types of soil surfaces, the migration process of diesel front was fast initially and then slowed down to reach a steady state in the soil (Figure 2). The migration rate of diesel before 0.5 h was about 1 cm/h, then gradually trended to 0. The previous study reported that NAPLs are strongly driven by gravity at the initial stage of leakage [37]. Compared with the migration distance of diesel in fourteen types of soils, the migration process of diesel could be divided into three categories (I: 3.0 cm < *S* < 5.0 cm, II: 5.0 cm < *S* < 7.0 cm, III: *S* > 8.0 cm). For example, the migration distance of diesel in seven types of soils including gray desert soil (Qinghai), yellow soil (Guizhou), red earth (Jiangxi and Hunan), paddy soil (Zhejiang and Jiangsu), and purple soil (Chongqing) ranged 3.0 cm to 5.0 cm.

Combined with the migration data of diesel, the third-order exponential equation (Equation (3)) was used to fit the dynamic process of diesel migration in fourteen types of soils [38].

*S* = *Y*_e_ − *A*e^(−t/a)^ − *B*e^(−t/b)^ − *C*e^(−t/c)^ (3)

where *S* is the migration distance of diesel front in the soil column, cm; *Y*_e_ represents the theoretical migration distance when diesel formed steady state in the soil column, cm; and other values are equation fitting parameters.

The migration kinetics could be satisfactorily described with the third-order exponential equation (*R*^2^ > 0.99), and the equilibrium migration distance ranged from 3.42 cm (Guizhou yellow soil) to 8.82 cm (Hainan sandy soil) (Table S6). The equilibrium migration distance of diesel in fourteen types of soils is as follows: sandy soil > sandy loam soil > sandy clay loam soil > clay loamy soil > sandy clay soil, which further confirmed that soil structure characteristics are key factors that lead to the migration distance of diesel under spill condition [38].

### 3.3. Residual Amount of Diesel in Fourteen Types of Soils

**Figure 3:**
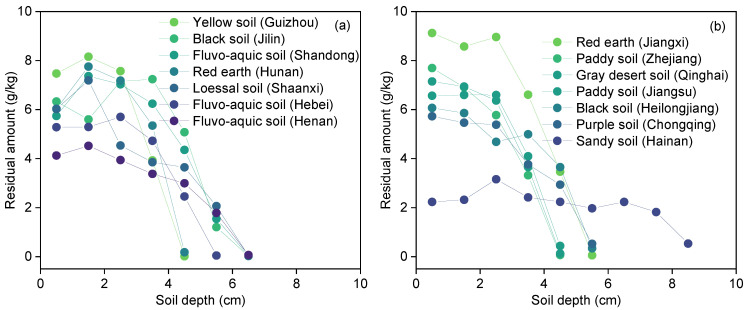
Residual amount of diesel in fourteen types of soils after 72 h.

Figure 3 showed the variation of the residual amount of diesel after migrating 72 h in fourteen types of soils. The residual amount of diesel in seven types of soils gradually increased first and then decreased with the increase in soil depth (Hunan red earth, Shandong fluvo-aquic soil, Shaanxi loessal soil, Guizhou yellow soil, Henan fluvo-aquic soil, Hebei fluvo-aquic soil, and Jilin black soil) (Figure 3a). For example, the maximum residual amount of diesel in Hunan red earth, Shandong fluvo-aquic soil, Shaanxi loessal soil, and Guizhou yellow soil were observed at 1.5 cm, the values were 7.8, 7.4, 7.2, and 8.2 g/kg, respectively. A similar phenomenon was reported by previous studies, which was attributed that the migration rate gradually decreased with the increase in soil depth [38,39]. Meanwhile, the high viscosity of diesel is also an important factor for its interception by the upper layer of soil [40]. As shown in Figure 3b, the residual amount of diesel in gray desert soil (Qinghai), purple soil (Chongqing), paddy soil (Zhejiang and Jiangsu), black soil (Heilongjiang), and red earth (Hunan) decreased with the increase in soil depth, even the residual amount of diesel decreased rapidly with the soil depth exceeding 2.5 cm. Due to the larger particle size and porosity, the residual amount of diesel is uniformly distributed in Hainan sandy soil. Since the shortest migration distance of diesel in fourteen types of soils was 3.45 cm, only the residual amount of diesel in the 0-3 cm soil layer were focused on in this study. In the 0–1 cm soil layer, the residual amount of diesel (*Y*_1_) ranged from 2.8 g/kg (Hainan sandy soil) to 7.7 g/kg (Zhejiang paddy soil). In the 1–2 cm soil layer, the residual amount of diesel (*Y*_2_) in nine types of soils is more than 6.0 g/kg, and the average concentration was 1.2 times that of diesel in the 0–1 cm soil layer. In summary, the residual amount of diesel in fourteen types of soils was as follows: sandy clay loam soil > clay loamy soil > sandy clay soil > sandy loam soil > sandy soil. 

### 3.4. Statistical Analysis of Soil Characteristics Affecting Diesel Migration and Residue 

Basic physiochemical properties and structure characteristics of soil could affect the behavior of NAPLs in the soil [11,41]. Herein, the basic physiochemical properties and structure characteristics of soil were selected as the independent variables of multivariate statistical analysis, and the main controlling factors affecting the migration distance and residual amount of diesel were analyzed by principal component regression and multiple linear regression analysis.

#### 3.4.1. Effect of Basic Physiochemical Properties of Soil

**Figure 4:**
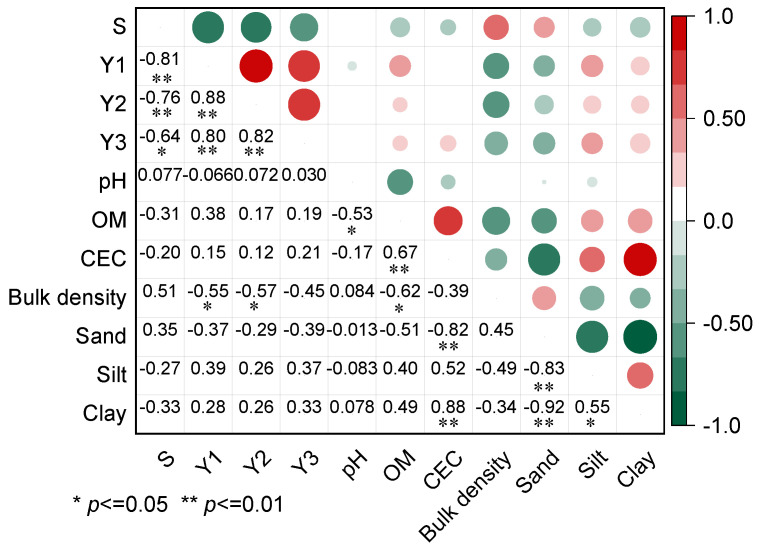
Correlation analysis between physiochemical properties, migration distance, and residual amount of diesel in the 0–3 cm soil layer.

Correlation analysis was conducted between the migration distance and residue amount of diesel in the 0–3 cm soil layer and the basic physicochemical properties of soil (Figure 4). Residual amounts of diesel in the 0–1 cm and 1–2 cm soil layers have a negative correlation with soil bulk density (*R*^2^ > 0.55, *p* ˂ 0.05). In comparison, there is a poor correlation between migration distance or residue amount of diesel in the 2–3 cm (*Y*_3_) soil layer and other basic physiochemical properties (e.g., organic matter content and cation exchange capacity). This result indicated that during the spill of diesel, the chemical properties of soil have little effect, while soil structure characteristics have an obvious effect on the migration distance and residue amount of diesel in unsaturated soil. The phenomenon was inconsistent with the current results that soil organic matter is a key factor in determining the adsorption of organic pollutants by soil [42,43], which was attributed to the fact that diesel with a fast infiltration rate is hard to fully interact with the soil [44]. Han et al. [20] found that although two types of soils with the same soil porosity and organic matter content, soil with high clay content possessed more micropores, resulting in the higher residual amount of NAPL in the soil.

#### 3.4.2. Effect of Soil Particle Size Distribution

As shown in Table S7, there was a negative correlation between migration distance and characteristic parameters of soil particle size distribution (except for specific surface area, diameter distance, and consistency of soil particle), and the residual amount of diesel in the 0-3 cm soil layer is negatively correlated with specific surface area of soil particle (*R*^2^ > 0.627, *p* < 0.01). Due to the multicollinearity between the characteristic parameters of soil particle size distribution (Table S2), we extract the principal components (*F*_1_ and *F*_2_) and then carry out a stepwise regression analysis with migration distance and residual amount of diesel. The regression equations are *S* = 5.201 + 1.074*F*_1_ − 0.574*F*_2_ (*R*^2^ = 0.722, *p* < 0.01), *Y*_1_ = 5.962 − 0.842*F*_1_ + 0.659*F*_2_ (*R*^2^ = 0.671, *p* < 0.01), *Y*_2_ = 6.355 − 1.014*F*_1_ (*R*^2^ = 0.493, *p* < 0.01), and *Y*_3_ = 5.901 – 0.96*F*_1_ (*R*^2^ = 0.433, *p* < 0.01), respectively.

The regression coefficients of standardized independent variables in the regression equation are listed in Table S8. It can be seen that in the regression equation of migration distance, the absolute value of the consistency coefficient is the largest (0.2004), indicating that the consistency is the main influencing factor on the migration distance of diesel in fourteen types of soils; similarly, the consistency has also the greatest influence on the residual amount of diesel in the 0–1 cm soil layer. The consistency is defined as the distribution uniformity of soil particle size distribution [45]. The larger the consistency value was, the stronger heterogeneity of soil pore size distribution and retardation to diesel migration, which reduced the migration ability of diesel [46], further increased the residual amount of diesel in the topsoil. In the regression equation of residual amounts of diesel in the 1–2 cm and 2–3 cm soil layers, the absolute value of Dv90 coefficient was 0.1415 and 0.1339, respectively, indicating that Dv90 has the greatest impact on the residual amounts of diesel in the 1–2 cm and 2–3 cm soil layer. Dv90 referred the soil particle size corresponding to 90% of the volume fraction [47]. The specific surface area of soil particle decreased as Dv90 value increased, which is adverse to the adsorption capacity of soil to diesel [48]. Moreover, the more soil pores formed by soil large particles could provide channels for diesel migration, resulting in the reduction in the residual amounts of diesel in 1–2 cm and 2–3 cm soil layers.

#### 3.4.3. Effect of Soil Pore Size Distribution

**Figure 5:**
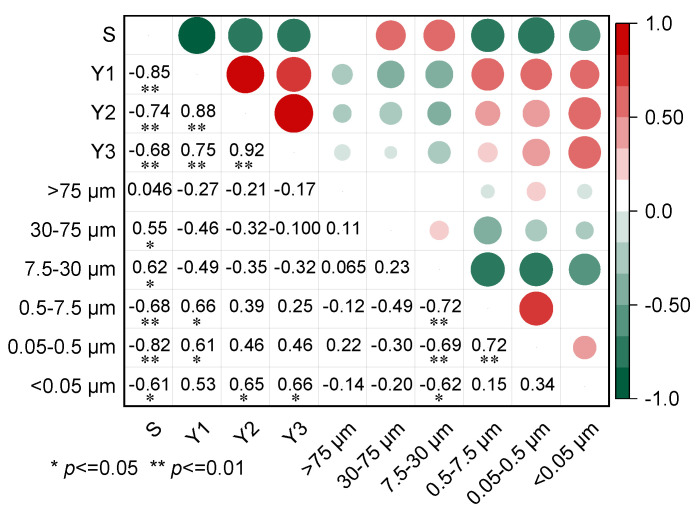
Correlation analysis between soil pore size distribution, migration distance, and residual amount of diesel in the 0–3 cm soil layer.

Figure 5 showed the correlation between migration distance and residual amount of diesel in the 0-3 cm soil layer and soil pore size distribution. Migration distance of diesel was positively correlated with the pore volume of soil pores at 30.0–75.0 μm (*R*^2^ = 0.55) and 7.5–30.0 μm (*R*^2^ = 0.62), but negative correlated with the pore volume of soil pores at 7.5–0.5 μm (*R*^2^ = 0.82), 0.5–7.5 μm (*R*^2^ = 0.68), and smaller than 0.05 μm (*R*^2^ = 0.61). It is known that the large pores contributed to reducing the capillary pressure that the NAPL need to overcome to go through these pores and thus migrate through the soil [10,49]. Similar results have been reported that soil macropores (>60 μm diameter) and mesopores (15 μm < diameter < 60 μm) was the main factor affecting the migration of fluid in the porous medium [15]. The correlation coefficient between the residual amount of diesel in the 0–1 cm soil layer and the pore volume of soil macropores (0.05–7.5 μm) was higher than 0.60, and the residual amount of diesel in the 1–3 cm soil layer was higher than the correlation with the pore volume of soil mesopores (<0.05 μm). The results indicated that soil macropores volume are considered to be important for the diesel residue on the soil surface, while the diesel residue under topsoil was affected by soil mesopores volume smaller than 0.05 μm significantly. The higher capillary force in the soil, which results from a small pore body and narrow throats, caused more diesel to be trapped in the pores [24].

## 4. Conclusions

In this study, the experiment simulated a diesel spill that was performed in fourteen types of soils distributed in the large-scale region of China to investigate the migration distance and vertical distribution of diesel. Based on statistical analysis, the key factors affecting diesel behavior are revealed in terms of soil particle and pore size distribution. There were significant differences between fourteen types of soils, with regard to the soil particle, soil pore, and diesel migration and residue. After 72 h of leakage, the migration distance of diesel ranged from 3.42 cm to 8.82 cm in fourteen types of soils. Except for sandy soil, diesel is mainly distributed in the 0–3 cm soil layer, and the residual amount were 7.85–26.66 g/kg, in the order of sandy clay loam soil > clay loamy soil > sandy clay soil > sandy loam soil > sandy soil. Further confirmed from macro-level that soil bulk density controlled the residue amount of diesel in the topsoil (0–2 cm). Soil microstructure including the consistency of soil particle and volume of soil macropores (0.05–7.5 μm) are considered to be important for the residual amount in the 0–1 cm soil layer and migration distance of diesel. The large soil particles corresponding to 90% of volume fraction and volume of soil mesopores (<0.05 μm) are key factors affecting the residual amount of diesel in the 1–3 cm soil layer. This study would provide support for investigating the formation process and mechanism of residual LNAPLs in the soil.

## Figures and Tables

**Table 1:** Physiochemical properties of fourteen types of soils.

Classification (Location)	pH	OM g/kg	CEC cmol/kg	Soil Bulk Density g/cm^3^	Sand %	Silt %	Clay %	Soil Texture
Gray desert soil (Qinghai)	7.90	23.10	5.81	1.3537	73.38	7.36	19.26	sandy clay loam soil
Yellow soil (Guizhou)	3.96	29.00	12.00	1.3537	82.03	2.80	15.17	sandy clay loam soil
Loessal soil (Shaanxi)	8.76	1.35	2.57	1.492	79.07	4.55	16.38	sandy clay loam soil
Fluvo-aquic soil (Hebei)	7.96	11.50	12.20	1.4669	60.47	17.02	22.51	sandy clay loam soil
Red earth (Jiangxi)	7.77	14.60	10.50	1.6052	63.41	13.08	23.51	sandy clay loam soil
Paddy soil (Zhejiang)	5.06	23.40	9.24	1.4040	59.99	26.62	13.39	clay loamy soil
Purple soil (Chongqing)	7.44	8.38	18.20	1.6219	66.69	5.65	27.66	clay loamy soil
Black soil (Jilin)	6.88	31.30	26.00	1.2951	33.06	26.71	40.23	clay loamy soil
Black soil (Heilongjiang)	5.55	47.90	34.90	1.3998	40.60	16.10	43.30	clay loamy soil
Red earth (Hunan)	7.69	7.46	21.20	1.5423	40.81	18.19	41.00	clay loamy soil
Fluvo-aquic soil (Henan)	7.98	13.60	9.35	1.5507	85.40	3.86	10.74	sandy loam soil
Fluvo-aquic soil (Shandong)	7.81	11.00	14.3	1.3789	74.84	11.76	13.40	sandy loam soil
Sandy soil (Hainan)	5.65	3.26	1.92	1.8734	94.30	0.80	4.90	sandy soil
Paddy soil (Jiangsu)	7.78	20.20	15.70	1.4878	64.34	10.33	25.33	sandy clay soil

**Table 2:** Characteristic parameters of soil particle size distribution of fourteen types of soils.

Classification (Location)	Diameter Distance	Consistency	SSA	D[3,2]	D[4,3]	Dv10	Dv30	Dv50	Dv60	Dv90	Cc	Cu
			m^2^/g	μm	μm	μm	μm	μm	μm	μm		
Gray desert soil (Qinghai)	4.06	2.08	1.03	5.85	45.61	2.36	8.54	18.27	25.37	76.54	10.76	1.22
Yellow soil (Guizhou)	4.62	1.40	2.32	2.59	9.21	0.98	2.48	4.96	6.98	23.92	7.16	0.90
Loessal soil (Shaanxi)	2.17	0.67	0.85	7.09	43.03	2.87	20.74	39.52	48.37	88.66	16.83	3.09
Fluvo-aquic soil (Hebei)	3.66	1.17	1.15	5.22	24.27	2.03	6.05	15.74	23.48	59.54	11.60	0.77
Red earth (Jiangxi)	4.73	2.54	1.67	3.60	25.65	1.36	4.09	8.66	12.31	42.37	9.06	1.00
Paddy soil (Zhejiang)	15.38	3.97	1.35	4.45	54.38	1.61	5.65	12.42	18.65	192.62	11.61	1.06
Purple soil (Chongqing)	5.71	1.80	1.66	3.62	19.00	1.34	3.75	8.62	12.94	50.59	9.69	0.81
Black soil (Jilin)	5.95	1.97	1.53	3.92	17.58	1.55	3.71	7.90	12.49	48.56	8.04	0.71
Black soil (Heilongjiang)	3.17	1.06	1.40	4.27	13.87	1.72	4.69	9.14	12.29	33.43	7.14	1.04
Red earth (Hunan)	5.27	1.69	1.91	3.14	12.49	1.27	3.07	5.78	8.03	31.73	6.31	0.92
Fluvo-aquic soil (Henan)	3.03	1.00	0.99	6.09	39.25	2.24	10.84	28.76	38.16	89.23	17.06	1.38
Fluvo-aquic soil (Shandong)	4.07	1.35	1.00	6.02	35.80	2.28	7.10	21.40	37.33	89.29	16.36	0.59
Sandy soil (Hainan)	2.16	0.65	0.39	15.53	303.27	10.13	160.31	281.98	342.58	619.32	33.81	7.41
Paddy soil (Jiangsu)	4.58	1.48	1.35	4.45	18.69	1.83	4.85	9.69	13.57	46.24	7.41	0.95

Note: SSA represents the specific surface area; D[3,2] represents the surface area average particle size; D[4,3] represents the volume average particle size.

## Data Availability

Not applicable.

## Supplementary Materials

The following supporting information can be downloaded at: https://www.mdpi.com/article/10.3390/toxics11010016/s1, Table S1: Correlation analysis between parameters of soil particle size distribution; Table S2: Multicollinearity diagnostic between soil particle size distribution parameters; Table S3: Eigenvalues and proportion of factor analysis; Table S4: Volume percentage of different soil pore tested by mercury intrusion porosimetry; Table S5: Volume of different soil pore tested by mercury intrusion porosimetry; Table S6: Fitted results of diesel transport kinetics by third-order exponential function; Table S7: Correlation analysis between parameters of soil particle size distribution, residual concentration and migration depth; Table S8: Principal component regression coefficients and constants.
