# Supplementary material for: Novel Insights into the Influence of Soil Microstructure Characteristics on the Migration and Residue of Light Non-Aqueous Phase Liquid
Source: Toxics. 2022 Dec 24;11(1):16. doi: 10.3390/toxics11010016 (PMC9863872; doi:10.3390/toxics11010016)
Supplement: Supplementary file 1 [file toxics-11-00016-s001.zip › toxics-2105370-supplementary.pdf]

Supplementary Materials: Novel Insights into the Influence of Soil Microstructure Characteristics on the Migration and Residue of Light Non-Aqueous Phase Liquid

Xiaodong Li, Qian Zhang, Xueli Zhang, Jialun Shen, Zongquan Sun, Fujun Ma, Bin Wu and Qingbao Gu

Table S1. Correlation analysis between parameters of soil particle size distribution

	**Diameter distance**	**Consistency**	**SSA**	**D[3,2]**	**D[4,3]**	**Dv10**	**Dv30**	**Dv50**	**Dv60**	**Dv90**	**Cc**	**Cu**
Diameter distance	1	**										
Consistency	0.908	1										
SSA	0.28	0.347	1	**	*	**	*	*	*	*	**	*
D[3,2]	−0.335	−0.429	−0.829	1	**	**	**	**	**	**	**	**
D[4,3]	−0.173	−0.258	−0.658	0.954	1	**	**	**	**	**	**	**
Dv10	−0.313	−0.402	−0.719	0.982	0.985	1	**	**	**	**	**	**
Dv30	−0.276	−0.367	−0.622	0.949	0.989	0.99	1	**	**	**	**	**
Dv50	−0.283	−0.377	−0.643	0.958	0.99	0.993	0.999	1	**	**	**	**
Dv60	−0.281	−0.376	−0.653	0.962	0.991	0.994	0.998	0.999	1	**	**	**
Dv90	−0.043	−0.164	−0.648	0.93	0.989	0.959	0.966	0.967	0.969	1	**	**
Cc	−0.28	−0.396	−0.808	0.964	0.912	0.927	0.897	0.913	0.92	0.902	1	**
Cu	−0.311	−0.41	−0.648	0.943	0.952	0.965	0.97	0.968	0.963	0.924	0.894	1

Note: Pearson correlation; * significant at 0.05 level; ** significant at 0.01 level.

Table S2. Multicollineatity diagnostic between soil particle size distribution parameters

Model dimension	Characteristic value	Conditional number	Variance ratio								
			Z_1	Z_2	Z_3	Z_7	Z_8	Z_9	Z_{10}	Z_{11}	Z_{12}
1	7.414	1.00	-	-	-	-	0.0001	0.0004	-	-	-
2	2.153	1.86	-	-	0.0001	-	-	0.0007	0.0001	0.0001	0.0002
3	0.290	5.05	0.0001	-	-	0.0001	0.0013	0.0019	0.0007	0.0013	0.0010
4	0.080	9.60	-	0.0001	0.0007	0.0005	0.0251	0.0115	0.0073	0.0001	-
5	0.030	15.73	0.0001	0.0003	0.0026	-	0.0039	0.6213	0.0013	0.0023	0.0006
6	0.024	17.72	0.0003	0.0003	0.0001	0.0002	0.0199	0.0254	0.0024	0.0126	0.0515
7	0.008	29.77	0.0001	-	0.0001	0.0085	0.3913	0.0490	0.0048	0.0033	0.0157
8	0.001	107.25	0.0158	0.0705	0.8324	0.0079	0.0011	0.0378	0.0637	0.0141	0.0698
9	-	192.06	0.2244	0.0208	0.1213	0.3716	0.0023	0.2517	0.2322	0.2412	0.0074
10	-	341.74	0.7593	0.9080	0.0428	.6112	0.5548	0.0003	0.6874	0.7250	0.8537
	VIF		3996.6	4145.5	838.8	832.2	33.2	21.4	78.9	251. 9	87.1

Note: “-” less than 0.0001.

Table S3. Eigenvalues and proportion of factor analysis

	Initial Characteristic Root			Rotation Characteristic Root		
	**Characteristic Root**	**Contribution of Variances/%**	**Cumulative Contribution/%**	**Characteristic Root**	**Contribution of Variances/%**	**Cumulative Contribution/%**
*Z*1	9.451	78.757	78.757	8.987	74.893	74.893
*Z*2	1.750	14.580	93.337	2.213	18.445	93.337
*Z*3	0.577	4.811	98.149			
*Z*4	0.101	0.842	98.991			
*Z*5	0.065	0.545	99.535			
*Z*6	0.052	0.432	99.967			
*Z*7	0.003	0.022	99.989			
*Z*8	0.001	0.009	99.998			
*Z*9	0	0.001	100			
*Z*10	0	0	100			
*Z*11	0	0	100			
*Z*12	0	0	100			

Table S4. Volume percentage of different soil pore tested by mercury intrusion porosimetry

Classification (location)	>75 μm	30–75 μm	7.5–30 μm	0.5–7.5μm	0.05–0.5 μm	< 0.05 μm
Gray dese soil (Qinghai)	4.06	1.79	21.88	42.16	17.62	12.49
Yellow soil (Guizhou)	4.32	4.42	22.17	34.89	22.63	11.57
Loessal soil (Shaanxi)	4.11	1.39	69.20	12.85	5.69	6.77
Fluvo-aquic soil (Hebei)	3.32	4.62	57.81	16.69	9.59	7.96
Red earth (Jiangxi)	4.86	4.11	37.34	13.33	11.84	28.50
Paddy soil (Zhejiang)	3.27	0.80	5.89	63.98	17.36	8.70
Purple soil (Chongqing)	4.66	6.35	41.10	22.61	16.53	8.76
Black soil (Jilin)	4.14	15.80	53.98	11.07	6.65	8.36
Black soil (Heilongjiang)	3.72	5.31	47.74	25.26	8.72	9.26
Red earth (Hunan)	5.11	6.90	22.06	27.27	15.56	23.10
Fluvo-aquic soil (Henan)	6.63	3.27	64.33	14.74	5.68	5.35
Fluvo-aquic soil (Shandong)	3.52	5.49	70.62	11.62	4.47	4.27
Sandy soil (Hainan)	6.02	21.29	57.79	4.42	3.35	7.14
Paddy soil (Jiangsu)	5.10	5.41	23.28	30.07	26.53	9.61

Table S5. Volume of different soil pore tested by mercury intrusion porosimetry

Classification (location)	>75 μm	30–75 μm	7.5–30 μm	0.5–7.5 μm	0.05–0.5 μm	<0.05 μm
Gray dese soil (Qinghai)	0.0093	0.0041	0.05	0.0965	0.0403	0.0285
Yellow soil (Guizhou)	0.0118	0.0122	0.0608	0.0956	0.0621	0.0317
Loessal soil (Shaanxi)	0.0095	0.0032	0.1597	0.0296	0.0131	0.0156
Fluvo-aquic soil (Hebei)	0.008	0.0111	0.1387	0.0401	0.023	0.0192
Red earth (Jiangxi)	0.0089	0.0075	0.0664	0.0259	0.0216	0.052
Paddy soil (Zhejiang)	0.0082	0.002	0.0149	0.161	0.0437	0.0219
Purple soil (Chongqing)	0.0106	0.0145	0.0936	0.0515	0.0376	0.0199
Black soil (Jilin)	0.0106	0.0407	0.1388	0.0284	0.0171	0.0215
Black soil (Heilongjiang)	0.0087	0.0124	0.1116	0.0591	0.0203	0.0217
Red earth (Hunan)	0.0102	0.0138	0.044	0.0545	0.031	0.0462
Fluvo-aquic soil (Henan)	0.0153	0.0075	0.1482	0.0340	0.0131	0.0123
Fluvo-aquic soil (Shandong)	0.0086	0.0134	0.1725	0.0284	0.0109	0.0104
Sandy soil (Hainan)	0.0102	0.0360	0.0978	0.0075	0.0057	0.0121
Paddy soil (Jiangsu)	0.0129	0.0136	0.0588	0.076	0.067	0.0243

Table S6. Fitted results of diesel transport kinetics by third-order exponential function

Soil types	Third-order exponential equation	R^2	Migration distance/cm
Gray desert soil (Qinghai)	y=4.26-1.14e$^{(-t/1.929)}$-1.50e$^{(-t/45.59)}$-1.60e$^{(-t/0.020)}$	0.9986	4.26
Yellow soil (Guizhou)	y=3.46-0.77e$^{(-t/0.993)}$-1.27e$^{(-t/24.87)}$-1.41e$^{(-t/0.014)}$	0.9984	3.46
Loessal soil (Shaanxi)	y=5.76-1.51e$^{(-t/1.078)}$-2.82e$^{(-t/23.02)}$-1.42e$^{(-t/0.016)}$	0.9961	5.76
Fluvo-aquic soil (Hebei)	y=5.16-1.86e$^{(-t/20.29)}$-1.14e$^{(-t/1.315)}$-2.14e$^{(-t/0.014)}$	0.9983	5.16
Red earth (Jiangxi)	y=4.42-1.85e$^{(-t/0.018)}$-0.77e$^{(-t/1.227)}$-1.78e$^{(-t/32.80)}$	0.9984	4.42
Paddy soil (Zhejiang)	y=3.98-1.34e$^{(-t/0.024)}$-0.85e$^{(-t/0.891)}$-1.76e$^{(-t/30.451)}$	0.9984	3.98
Purple soil (Chongqing)	y=4.57-2.02e$^{(-t/0.027)}$-0.94e$^{(-t/1.238)}$-1.57e$^{(-t/40.549)}$	0.9978	4.57
Black soil (Jilin)	y=5.94-2.06e$^{(-t/0.331)}$-2.42e$^{(-t/16.67)}$-1.45e$^{(-t/0.017)}$	0.9962	5.94
Black soil (Heilongjiang)	y=5.49-1.41e$^{(-t/1.364)}$-2.35e$^{(-t/0.022)}$-1.70e$^{(-t/31.67)}$	0.9984	5.49
Red earth (Hunan)	y=4.23-0.94e$^{(-t/1.629)}$-1.47e$^{(-t/39.47)}$-1.81e$^{(-t/0.024)}$	0.9991	4.23
Fluvo-aquic soil (Henan)	y=6.52-1.53e$^{(-t/1.262)}$-3.22e$^{(-t/30.34)}$-1.74e$^{(-t/0.017)}$	0.9974	6.52
Fluvo-aquic soil (Shandong)	y=6.43-1.83e$^{(-t/37.14)}$-2.29e$^{(-t/2.622)}$-2.26e$^{(-t/0.061)}$	0.9988	6.43
Sandy soil (Hainan)	y=8.82-2.66e$^{(-t/1.24)}$-2.52e$^{(-t/0.017)}$ -3.62e$^{(-t/36.093)}$	0.9989	8.82
Paddy soil (Jiangsu)	y=3.77-1.53e$^{(-t/0.031)}$-1.01e$^{(-t/1.504)}$-1.21e$^{(-t/23.663)}$	0.9991	3.77

Table S7. Correlation analysis between parameters of soil particle size distribution, residual concentration and migration depth

Characteristic parameters	Independent variable	Y_1(0–1 cm)	Y_2(1–2 cm)	Y_3(2–3 cm)	S
Dv90	Z_1	−0.627*	−0.684**	−0.633*	0.717**
D[4,3]	Z_2	−0.666**	−0.694**	−0.630*	0.743**
Dv60	Z_3	−0.723**	−0.717**	−0.642*	0.785**
Dv10	Z_4	−0.726**	−0.736**	−0.670**	0.798**
Dv50	Z_5	−0.718**	−0.717**	−0.646*	0.776**
Dv30	Z_6	−0.702**	−0.705**	−0.635*	0.758**
D[3,2]	Z_7	−0.739**	−0.766**	−0.725**	0.838**
Cu	Z_8	−0.746**	−0.748**	−0.721**	0.859**
Cc	Z_9	−0.646*	−0.691**	−0.693**	0.730**
SSA	Z_{10}	0.627*	0.721**	0.725**	−0.763**
Diameter distance	Z_{11}	0.525	0.321	0.254	−0.438
Consistency	Z_{12}	0.647*	0.487	0.456	−0.542*

* significant at 0.05 level; ** significant at 0.01 level.

Table S8. Principal component regression coefficients and constants

Characteristic parameters	S	Y_1	Y_2	Y_3
Dv90	0.0685	−0.0241	−0.1415	−0.1339
D[4,3]	0.0906	−0.0546	−0.1282	−0.1213
Dv60	0.1119	−0.0850	−0.1127	−0.1067
Dv10	0.1188	−0.0946	-0.1084	−0.1026
Dv50	0.1119	−0.0852	−0.1122	−0.1063
Dv30	0.1098	−0.0825	−0.1131	−0.1070
D[3,2]	0.1253	−0.1046	−0.1018	−0.0964
Cc	0.1175	−0.0949	−0.1037	−0.0982
Cu	0.1166	−0.0941	−0.1031	−0.0976
SSA	-0.1062	0.0961	0.0671	0.0636
Diameter distance	-0.1957	0.2614	−0.0952	−0.0901
Consistency	-0.2004	0.2615	−0.0788	−0.0746
Constants	5.2010	5.9620	6.3550	5.9010
